# Thyroid-like follicular carcinoma of the kidney in a patient with nephrolithiasis and polycystic kidney disease: a case report

**DOI:** 10.1186/1746-1596-8-108

**Published:** 2013-07-02

**Authors:** Metka Volavšek, Margareta Strojan-Fležar, Gregor Mikuz

**Affiliations:** 1 Institute of Pathology, Faculty of Medicine, University of Ljubljana, Korytkova 2, 1000, Ljubljana, Slovenia; 2 Department of Pathology, University of Innsbruck, Innsbruck, Austria

**Keywords:** Renal cell carcinoma, Thyroid-like follicular carcinoma, Pathology-cytology, Immunohistochemistry, Polycystic kidney disease

## Abstract

**Abstract:**

Thyroid-like follicular carcinoma of the kidney (TLFC), a rare neoplasm with low malignant potential, is histologically similar to primary thyroid follicular carcinoma, but characteristically lacks thyroid immunohistochemical markers. We report a case of 34-year old patient with nephrolithiasis. Ultrasound revealed hepatorenal cysts consistent with adult type polycystic kidney disease (ATPKD) and a cytologically confirmed left kidney tumor. Nephrectomy specimen contained sharply demarcated lesion of unusual morphology. Tubular and cystic structures lined by mostly cuboidal cells and filled with amorphous eosinophillic material, reminiscent of follicular carcinoma of the thyroid gland, were diagnostic for TLFC. Thyroid markers were negative. To our knowledge this is the first report of TFLC associated to ATPKD. Brief review of previously published TFLCs, possible relationship between entities and differential diagnosis are discussed.

**Virtual slides:**

The virtual slide(s) for this article can be found here: http://www.diagnosticpathology.diagnomx.eu/vs/8067946569612694

## Background

Renal tumors which became unique clinico-pathologic entities with distinctive immunohistochemical, cytogenetic and molecular profiles have been successfully incorporated into the current WHO classification [1], which became the cornerstone of diagnostic work in the surgical pathology. Developments in the last decade have, however, led to description of additional entities with distinctive morphology [2], which are not yet covered in the current WHO classification. It is important to be aware of the existence of these new rare renal tumor entities because the description of further cases is needed in order to gain additional knowledge of their biological behaviour.

One of those new entities is the thyroid-like follicular renal cell carcinoma (TLFC) [2]. This rare neoplasm with low malignant potential is histologically similar to primary thyroid follicular carcinoma, but is characteristically negative for thyroid immunohistochemical markers. We describe a case of TFLC diagnosed in a patient with polycystic kidney disease.

## Case presentation

### Cinical history

A 34-year old patient has come to medical attention because of nephrolithiasis, proven by effective spontaneous stone elimination. Owing to persistence of the pain, the abdominal ultrasound (US) examination has been performed, revealing hepatorenal polycystic disease, consistent with adult type polycystic kidney disease (ATPKD) [3]. Patient had no familial history of ATPKD. Additionally, a hyperechogenic cyst has been discovered in his left kidney, measuring 4-5 cm in greatest diameter. US guided fine needle aspiration biopsy (US-FNAB) of the lesion confirmed a neoplasm and was followed by surgical removal of the left kidney. Systematic clinical examinations, including accurate imaging techniques, revealed no other tumors or disease changes. Renal function and blood pressure were normal and the results of urine laboratory examinations showed no abnormalities. Except for mild left lumbal pain persisting at the time of discharge from the hospital, the patient is well after 6 months, without any other signs of disease.

## Materials and methods

### Ultrasound-guided fine needle aspiration biopsy (US-FNAB)

Ultrasound-guided fine needle aspiration biopsy (US-FNAB) of atypical renal cyst was conducted by a radiologist using a 22-gauge needle attached to a 10-ml syringe. Direct smears were prepared on site, air dried and subsequently Giemsa stained. Few drops of blood stained fluid were submitted in the syringe. The material left in the syringe was suspended in short term storage cell medium. The cell suspension was used to prepare 10 cytospins using a Shandon cytospin 4 cytocentrifuge (Thermo Shandon Inc, USA). Cytopsins were immediately fixed in methanol. Test cytospins were stained according to Papanicolaou method. Immunostaining was performed in the automated immunostaining system NexES (Ventana Medical Systems Inc., USA). Bound primary antibodies were detected using an iView detection kit (Ventana Medical Systems Inc., USA). Immunocytochemical staining for RCC (renal cell carcinoma) marker (PN-15, Cell Marque), vimentin (clone V9, DAKO), CK7 (clone OV-TL 12/30, DAKO), P504S (α-methylacyl-CoA racemase-AMACR; clone 13H4, DAKO), CD 10 (clone 56C6, NCL) were performed.

### Nephrectomy

**Figure 1:**
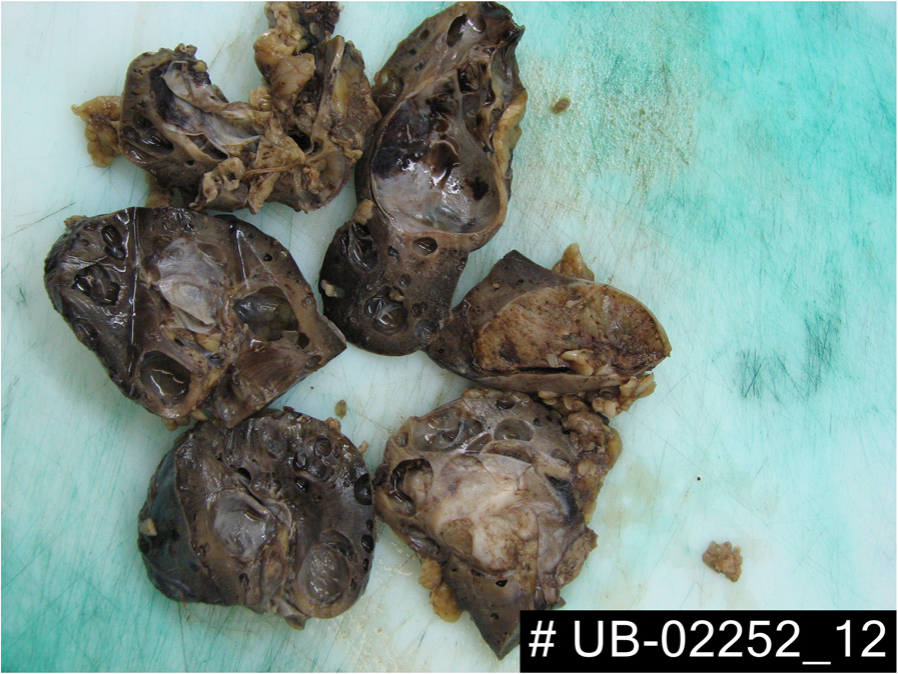
Polycystic left kidney demonstrating a 5.5×4.8 cm incapsulated, partially haemorrhagic and cystically degenerated solid grey tan tumor.

The left nephrectomy specimen including perinephric fat weighted 720 g and measured 18×13 cm in greatest diameters, the kidney measured 13×7 cm. Renal parenchyma was for the most part polycystic, with cysts measuring up to 4 cm in greatest diameter. Cyst walls were smooth and glistening, the cysts were filled with clear watery fluid. The sharply circumscribed encapsulated solid grey tan tumor was present in the lower pole of the kidney, measuring 5.5×4.8 cm (Figure 1). Centrally it was partially haemorrhagic and cystically degenerated. Formalin fixed representative tissue specimens, sampled according to the current protocols [4] were routinely stained with hematoxylin and eosin (HE). Additional immunohistochemical stainings were performed in a Ventana XT apparatus (Ventana Medical Systems Inc., USA), with automated staining procedures. The panel of primary antibodies included (source, clone and solutions): CAM5.2 (BECT. DICK., RTU), CD10 (NCL, 56C6, 1:15), CD15 (NCL, BY87, 1:20), CD56 (NCL, 1B6, 1:25), CD117 (DAKO, rb, 1:40), CEA (DAKO, rb, 1:1000), CK7 (DAKO, OV-TL 12/30, 1:100), CK20 (DAKO, Ks20.8, 1:20), CK34βe12 (ENZO, 34βe12, 1:50), CK AE1/AE3 (DAKO, AE1/AE3, 1:50), EMA (DAKO, E29, 1:20), P504S (DAKO, 13H4, 1:20), RCC (VENTANA, ready to use-RTU), TFE3 (CELL MARQUE clone MRQ-37; 1:100), Thyroglobulin (DAKO, rb, 1:5000), TTF-1 (DAKO, 8g7g3/1, 1:20), vimentin (DAKO, V9, 1:300),WT-1 (DAKO, 6 F-H2, 1:40). When needed, appropriate positive and negative controls have been used.

## Results

**Figure 2:**
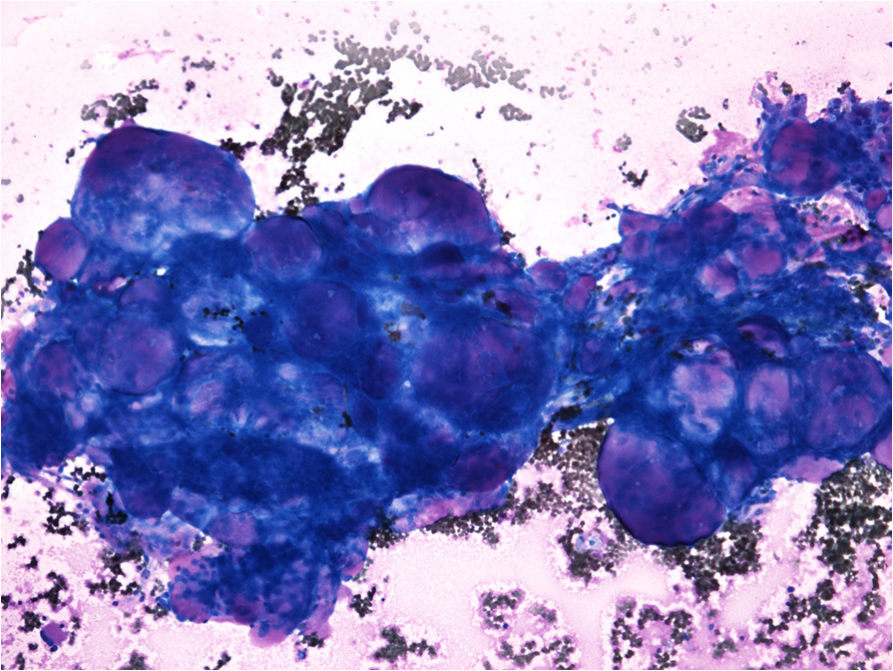
FNAB consisting of three-dimensional tissue fragments with numerous deep pink stained globules of different sizes (Giemsa, orig. magnif. ×10).

**Figure 3:**
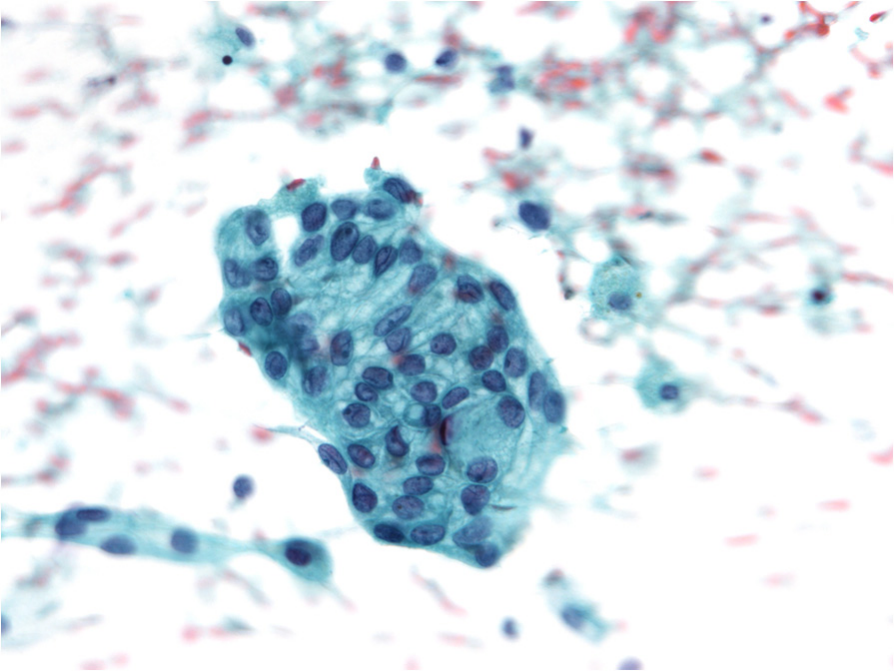
**A cluster of rather uniform medium sized cells in the FNAB smear.** Nuclei with small nucleoli are oval, with slight variation in size, some of them exhibiting grooves. The cytoplasm is moderate or more abundant, vacuolated, cell borders can be appreciated (Papanicoulaou, orig. magnif. ×40).

**US-FNAB** smear was highly cellular, the predominant morphological pattern were three-dimensional tissue fragments. Rounded papillary-like structures and nuclear pallisading was noted surrounding the edge of many fragments, while some exhibited cribriform-like pattern. In all, numerous deep pink stained globules of different sizes were seen (Figure 2). Calcifications were noted on the top of some particles. Cells were rather uniform, medium sized, mainly organized around pink globules. Nuclei were oval with slight variation in size, bland chromatin, with small nucleoli, some exhibiting grooves (Figure 3). The cytoplasm was moderate or more abundant, vacuolated, cell borders could be appreciated in Papanicolaou stained cytopins (Figure 3). Single cells were very rare, however some round naked nuclei were found. In the background there was abundant granulated pink material in addition to numerous macrophages and erythrocytes (Figure 2). Tumor cells were negative for RCC and CD10. They were positive for P504S, vimentin and CK7.

The proposed **cytological diagnosis** was papillary renal cell neoplasm, morphologically corresponding to MiTF/TFE family translocation-associated renal carcinoma.

**Figure 4:**
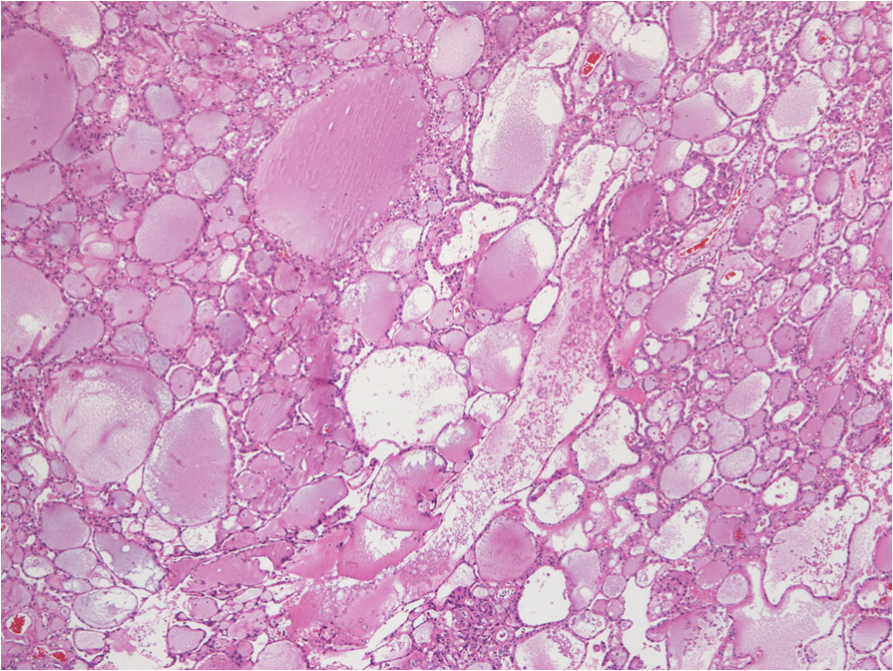
Follicles of various sizes, the dominant morphologic feature in histology, are lined by cuboidal cells and filled with partially colloid-like, partially mucoid material (HE, orig. magnif. x4).

**Figure 5:**
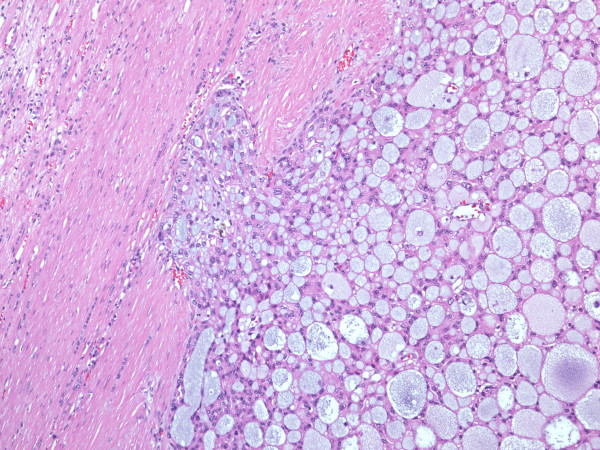
Invasion into the capsule, simulating capsular invasion of follicular thyroid carcinoma (HE, orig. magnif. ×10).

**Figure 6:**
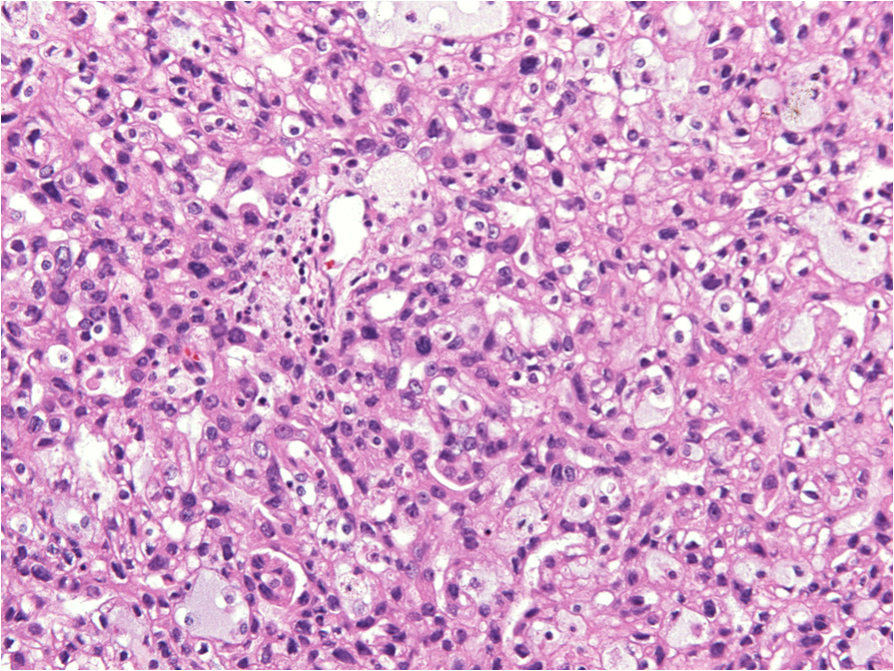
Cells with higher nuclear grade in more solid areas of the tumor (HE, orig. magnif. x20).

**Histologically**, the encapsulated tumor consisted of tubular and cytic structures reminiscent of follicular carcinoma of the thyroid gland. The structures lined by mostly cuboidal cells were filled with amorphous eosinophillic/basophillic material (partially colloid-like, partially mucoid; Figure 4). In some areas, the material extravasated out of the follicles into the stroma. The follicles varied in size, the nuclei were round to oval, the nucleoli only focally incospicuous, the chromatin was evenly distributed. Occasionally, nucleoli were more prominent, with nuclear features corresponding mostly to Fuhrman grade 2, focally even grade 3. Similarly to FNAB nuclear grooves could be detected in some areas**,** but there were no calcifications. Invasion into the capsule (Figure 5), resembling incipient but, since the tumor did not extend across the whole thickness of the capsule, not diagnostic capsular invasion of follicular thyroid carcinoma, was obvious on multiple locations at the periphery of the tumor. There was no mitotic activity. In some areas the tumor showed some papillary growth, focally it was even more solid (Figure 6). Those solid areas contained cells with higher nuclear grade comparing to macrofollicular areas.

In the nonneoplastic kidney, simple cysts were irregularly distributed, with focal signs of mild primary urine stasis in the Bowmann capsular space of the adjacent glomeruli. No other obvious chronic glomerular, vascular, or tubulointerstitial changes of the renal parenchyma could be seen, which is in accordance with clinically normal renal function. The only renal pathology, worth mentioning, was the presence of rare birefringent calcium oxalate crystals with small foci of adjacent mild chronic inflammatory infiltrate, confirming clinical picture of nephrocalculosis.

According to morphology and results of immunohistochemistry (Table 1), the histological diagnosis of TFLC was made.

**Table 1:** Immunohistochemical profile of the renal tumour

**Antibody**	**Result**
CAM5.2	positive
CD10	negative
CD15	negative
CD56	negative
CD117	negative
CEA	negative
CK7	positive
CK20	negative
CK34βe12	positive
CK AE1/AE3	positive
EMA	positive
P504S	positive
RCC	negative
TFE3	negative
thyroglobulin	negative
TTF-1	negative
vimentin	positive
WT1	negative

## Discussion

Thyroidization of the kidney is a well known phenomenon, where dilated tubular structures with atrophic epithelium containing colloid-like material imitate the usual structure of the thyroid gland. Usually, thyroidization occurs as a process secondary to chronic pyelonephritis and is a habitual characteristic of an end stage kidney disease. But the similarities between the kidney and the thyroid gland do not end up here. In the last years, a unique tumor type, primary to the kidney, but essentially looking as a thyroid lesion has been described, being named TLFC of the kidney. The first and by now the only real series consisted of 6 cases [5], 4 of those tumors being described for the second time by the same author. The well circumscribed neoplasms histologically resembling follicular carcinoma of the thyroid gland, but lacking typical thyroid markers, were reported to have favorable prognosis. Only one of the patients from that series, with tumors measuring up to 11.8 cm in greatest diameter, developed a metastasis in the renal hilar lymph node. The other patients were disease free in the follow up period from 7 to 84 months [5].

Additional case reports appeared in the literature [6-10], all but one describing tumors with follicular morphology, composed of cells with moderate amphophilic to slightly eosinophilic cytoplasm creating macro and microfollicles containing inspissated colloid-like material, with only small amount of packed follicles devoid of secretions. One of two cases described by Alessandrini et al. [9], however showed focal papillary arhitecture, without nuclear grooving or optical clearing. In our case, nuclear grooves have been visible in both specimens. In FNAB, focal calcifications were additionally noted on top of some fragments, which was not the case in histological samples, where they were absent. Analogous to our case, nuclear grooves in FNAB of TFLC have been noted by Dhillon et al. [11]. The authors retrospectively described FNAB results after already publishing histologic and clinical characteristics of the same TFLC, unique for the presence of lung and retroperitoneal lymph nodes metastases [8]. This was also the only case of TFLC with distant metastases, as these tumors generally have low malignant potential [12].

Concerning the tumor growth pattern in our case, although predominantly macrofollicular in HE slides, the papillary tumor growth could be observed focally in the histology, as well as in FNAB. This, in addition to calcifications present in FNAB, has led to the cytological diagnosis of papillary renal cell neoplasm, morphologically corresponding to MiTF/TFE family translocation-associated renal carcinoma, which was subsequently excluded by morphology of the nephrectomy specimen and negative TFE3 immunohistochemistry.

Marked lymphocytic infitration may be present in cases of TFLC, mostly as prominent intratumoral collections, but sometimes it is seen at the periphery, surrounding the tumor. These collections may occasionally contain lymphoid follicles with reactive germinal centers [12]. Our case was devoid of inflammation. Due to the presence of calcium oxalate crystals, only mild lymphocytic infiltrate was present around focally disrupted tubuli in the medulla.

Immunohistochemically, the TFLCs described in the literature showed variable, although relatively consistent negativity for Pax-2, RCC, CD10, WT-1, P504S, vimentin, CD56 and CD57 [12], and typical (but not obligate) negativity for CK7. For the diagnosis of TFLC all cases should be negative for thyroid markers such as TTF-1, thyroglobulin and galectin-3. In one reported case [13] the tumor was thyroglobulin positive, but the authors did not test TTF-1 or galectin-3. The exclusion of a metastazing thyroid carcinoma was carried out merely with clinical methods therefore one can argue, that the kidney tumor was a metastasis. However, the uneventful follow up of 18 months is a strong argument against a metastasizing thyroid carcinoma.

Although very rare, metastases to the kidney from the thyroid have been reported. Due to typical follicular morphology of the FTCL the metastasis of follicular thyroid carcinoma should be excluded in the first place. Metastatic follicular and papillary thyroid carcinoma in the kidney usually occur in patients with disseminated disease [14,15], sometimes even long after the primary tumor has been treated [16,17]. Another possible origin of a TTF-1 positive follicular tumor metastatis is struma ovarii, which is a very remote possibility, as are other follicular neoplasms, entering differential diagnosis such as serous and small cell carcinoma of the ovary, intrahepatic cholangiocarcinoma or breast tumors with follicular morphology [5,9]. Other rare primary tumors of the kidney with follicular growth pattern include carcinoid of the kidney (our case was CD56 negative) and, due to thyroidization-like appearance, the acquired cystic-disease associated RCC, which will be discussed later.

Returning to discussion on immunohistochemistry, the present TTF-1 and thyroglobulin negative case fits into the criteria for TLFC. However, diffuse positivity for CK7 and P504S was detected. Since in the literature variable staining results for those two markers were reported, they do not exclude our diagnosis «per se«. Nevertheless, our immunohistochemical results open the question whether or not this tumor could be a variant of papillary RCC. The only FISH analysis [7], which was performed on this tumor, did not show the clasical aberrations of the papillary carcinoma (trisomy 3q, 7, 8, 12, 16, 17 and loss of Y chromosome) but completely unspecific aberrations (loss of chromosomes 1, 3, 7, 9p21, 12, 17, and X). Genetic profiling of RCCs generally helps defining renal cell tumor subtypes in cases with inconclusive morphology, as shown in a report of synchronous clear cell RCC and tubulocystic carcinoma [18]. However, more data on genetic changes are needed to draw conclusions and clarify differential diagnosis in cases of TFLC.

Due to thyroidization like appearance of TFLC, the acquired cystic-disease associated RCC is another differential diagnosis. However, our patient did not suffer from an acquired but a hereditary renal cystic disease, which is the most common hereditary renal cystic disease. The clinical deterioration of the renal function can go unnoticed and the disease is diagnosed as an incidental finding at autopsy. Mostly, the end stage kidney disease appears at the age of 40 years. In our patient the polycystic disease has been diagnosed incidentally at the US examination due to persistent pain after spontaneous renal stone elimination. Although the patient is not an at-risk individual (no ADPKD familal history) the diagnosis of ADPKD was made clinically according to US criteria, since it is well known, that the family history may be absent in 20-40% patients [1]. Molecular analysis for determination of patient's genetic status was not performed, neither was it required for the diagnosis. As already mentioned, the patient's kidney function has not yet deteriorated, his blood pressure is normal.

Cystic appearance of the kidney containing solid tumor leads to differential diagnostic consideration of another rare primary renal neoplasm with favourable prognosis, namely mixed epithelial stromal tumor of the kidney (MEST), tumor with morphological similarities to cystic nephroma. However, MEST contains ovarian type stroma positive for estrogen and progesterone receptors and is predominantly observed in middle aged women [19].

The association between polycystic kidney disease and RCC has been well documented [20-22], as well as the association of acquired polycytic kidney disease with renal tumors, which now represent a special type of tumor, already included into the WHO classification [1]. One of the characteristics of those tumors, in addition to the end stage kidney disease, is the abundance of calcium oxalate crystals. Similar calculi, although not so abundant, have been found also in the kidney of our patient. In the first place, they were the reason for the diagnosis of ADPKD as well as of renal tumor, since the presenting symptom in our patient has been nephrolithasis with spontaneous stone elimination. However, no specific type of neoplasm has been descibed to occur in ADPKD. Most common histological subtypes assocated with ADPKD were clear cell RCC, followed by papillary and tubulocystic carcinoma [20-22]. Primary neuroendocrine tumors of the kidney, although rare, have also been reported to most commonly arise in the setting of acquired and congenital abnormalities, including polycystic kidney disease in 2% [23]. We are not aware of any published case of FTLC occuring in association with ADPKD. Our case, however shares some characteristics of papillary RCC, for instance CK7 and P504S positivity as well as focal papillary growth. Clinicaly, the association to ADPKD would also make more sense if the diagnosis in our case would be papillary RCC. However, the macrofollicular morphology, inspissated material, RCC and CD10 negativity as well as CK34βe12 positivity are the hallmarks of TLFC. There was no difference between follicular and papillary areas in the immunostaining patterns. Thyroid like kidney tumors with both, follicular and papillary features have been described also by other authors [24-26].

## Conclusions

In conclusion, this is the first case of TFLC associated with ADPKD. The possible correlation to cystic kidney disease has already been confirmed for RCC and other renal rumors, but should be further elucidated for TLFC. Moreover it seems, that the predominant pattern of thyroid like renal cancer is follicular indeed, but papillary features can be encountered as well. Future investigations are needed to verify whether this tumor is a true new nosologic entity or only a variant of one of the conventional RCCs.

### Consent

Written informed consent was obtained from the patient for publication of this Case Report and any accompanying images. A copy of Written concent is available for review by the Editor-in Chief of this Journal.

## Competing interests

The authors declare they have no competing interests.

## Authors’ contributions

MV carried out the morphological analyses and wrote the manuscript. MSF performed the cytological examination, GM participated in design and helped to draft the manuscript. All authors read and approved the final manuscript.

## Authors’ information

MV is Head of the Department of Uropathology, MSF is Head of Department of Cytopathology, both Institute od Pathology, Faculty of Medicine, University of Ljubljana, SLOVENIA. GM is former Head of Department of Pathology, University of Innsbruck, AUSTRIA and a former Chair of the Working group for Uropathology of the European Society of Pathology.
